# The Effects of Discharge Plan on Stress, Anxiety and Depression in Patients Undergoing Percutaneous Transluminal Coronary Angioplasty: A Randomized Controlled Trial

**Published:** 2014-04

**Authors:** Farkhondeh Sharif, Fariba Moshkelgosha, Zahra Molazem, Majid Najafi Kalyani, Mehrdad Vossughi

**Affiliations:** 1 Shiraz Geriatric Research Center, School of Nursing and Midwifery, Shiraz University of Medical Sciences, Shiraz, Iran;; 2 Department of Postgraduate Studies, School of Nursing and Midwifery, Shiraz University of Medical Sciences, Shiraz, Iran;; 3 Community Based Psychiatric Care Research, Department of Postgraduate Studies, School of Nursing and Midwifery, Shiraz University of Medical Sciences, Shiraz, Iran;; 4 Department of Dental Public Health, School of Dentistry, Shiraz University of Medical Sciences, Shiraz, Iran

**Keywords:** Discharge Plan, Angioplasty, Stress, Anxiety, Depression

## Abstract

**Background:** Angioplasty is one of the most common methods for treating coronary artery diseases. However, a large number of those undergoing this treatment face psychological problems that negatively affect the quality of their life and recovery. We aimed to determine the effects of discharge planning on stress, anxiety, and depression in patients undergone percutaneous transluminal coronary angioplasty (PTCA).

**Methods:** In this randomized controlled trial, 80 candidates for PTCA during January to April 2013 were randomly assigned to equal experimental and control groups. The patients in the experimental group participated in two training sessions before and after the procedure and an informative booklet was used for their training. These patients were followed by phone during the two weeks after discharge. The depression anxiety stress scale (DASS-21) was completed by all subjects upon admission, at discharge, and one month after discharge. Data were analyzed using SPSS software, version 18. *t* test was used as appropriated.

**Results: **The experimental group showed a statistically significant decrease in their stress, anxiety and depression a month after receiving the planned discharge (P<0.001). Although scores of stress (P=0.696), anxiety (P=0.110), depression (P=0.073) of the experimental group did not differ significantly on the day of discharge, the decrease was considerable compared with that of the control group.

**Conclusion:** Using a planned discharge program in patients undergoing PTCA lowered their stress, anxiety, and depression.

**Trial Registration Number: **IRCT201302182812N12

## Introduction

Cardiovascular diseases (CVD) are among the most important causes of mortality worldwide.^1^ Among them, coronary artery disease (CAD) is the most common cause of mortality.^2^ Similar to developed countries, evidence indicates an increased prevalence of CAD in Iran.^1^^-^^3^ Although the number of deaths due to CAD in developed countries is mostly seen in people over 70 years of age, in Iran, it is usually observed among those younger than this age.^2^ To treat CAD, different methods including coronary bypass, drug therapy, and percutaneous transluminal coronary angioplasty (PTCA) have been used; among which, PTCA is the most common.^4^

PTCA is considered as a potentially stressful event in patients’ lives, owing to the fact that it is not only an invasive procedure leading to a life threatening status, but it also necessitates an change in the individual’s lifestyle.^5^ These patients are more likely to confront psychological problems since their hospital stay is short and therefore, they do not have adequate access to those who might be able to provide them with psychological support.^6^

For various reasons, stress, anxiety, and depression are factors that negatively affect the patients’ recovery and quality of life. Myocardial infarctions and sudden death is 3 to 6 times more in anxious patients compared to the normal population.^6^^,^^7^ Most of the time those who are stressed, anxious and depressed show severe systematic reactions to stressors and experience an elevated heart rate and blood pressure as a result of catecholamine release. As a result, the hearts demand for blood and oxygen and lead to severe ischaemia.^8^^,^^9^ Moreover, in depressed patients, the incidence of hypertension,^10^ diabetes mellitus,^11^ and hyperlipidemia,^12^ each of which is considered as a risk factor for cardiac disease, is higher than the normal population. In addition, depressed mood is an independent risk factor for cardiac disease^13^ and mortality in patients with CAD.^11^^-^^14^ Stress and anxiety delay the period of adjustment to cardiac disease,^15^ and negatively affect the patients’ quality of life.^16^^-^^18^ Anxious patients are less able to adjust with the change of their lifestyle and diet, adhere to administered drugs and treatment, and regulate the amount of appropriate physical activities.^19^ Moreover, it anxiety persists it would lead to the severity of signs and symptoms, more physical disability, poor performance, and postponing the preparation for returning to their jobs and usual social activities.^20^

Basically, any significant change or poor performance requires physical, social, and psychological adjustment. In this regard, the hospital is regarded as a safe and supportive place for hospitalized patients, but the word “discharge” itself is threatening and stressful for them. As a matter of fact, the patients are concerned about their discharge and are preoccupied about their ability in performing their own duties and the way to handle themselves as well as joining the family.^21^ Such worries might be caused by the lack of sufficient information about caring for themselves after discharge.^22^ At discharge, less than half of the patients know nothing about their definite diagnosis, medication, the reason to take medication, and their main side effects.^23^ Moreover, almost one fourth of the patients encounter adverse consequences a month after discharge, most of which are preventable through training.^24^^,^^25^ Therefore, self-care training is of utmost importance for the patients and their families.^22^

Nurses, as key members of the treatment team, play a critical role in training and taking care of the patients. One of the most basic nursing responsibilities is to provide continuous care. In this regard, the inclusion of a discharge plan for all admitted patients could be a symbol of such care.^26^ In fact, the discharge plan begins at the admission time and includes the patients and their families’ needs prediction, and a plan to fulfill their requirements after discharge from hospital. A practical discharge plan helps to provide a continuous care with the least amount of stress for patients.^26^ In spite of the benefits of the discharge plan and its record in Europe and the US, it is still a controversial issue.^26^ However, few studies have been done on the discharge plan in Iran and the consequences of its performance are not clear yet. Upon discharge, patients undergoing PTCA require a lot of training regarding lifestyle change, wound care, the amount of physical activities, diet and their medications.^27^^,^^28^ However, it has been observed that these patients have been discharged without any needs assessment and no principles exist about the continuation of care at home. Moreover, because of workload and forgetfulness, nurses usually do not mention essential issues about treatment or they might be reminded at the very last moments of the patients’ discharge which hardly ever provide the continuance of the care. Therefore, their discharge issue should be revised. Moreover, it is claimed that these patients are more likely to experience stress, anxiety and depression^6^ which not only cause the rejection of cardiac rehabilitation,^29^ but also negatively affect their recovery and quality of life.^9^^,^^11^^,^^30^ On the other hand, the rejection of cardiac rehabilitation and the deterioration of disease increases stress and anxiety.^31^ Therefore, new methods should be used to remove or stop the vicious circle between the ailment and its consequences.

Considering the lack of sufficient studies in this field and its significance for patients undergoing angioplasty, we aimed to investigate the effects of discharge plan on the emergence of psychological factors caused by PTCA, using a practical discharge plan as a non-pharmacological and inexpensive therapy.

## Materials and Methods

This randomized controlled trial was done during January to April 2013 at three hospitals (Al-Zahra, Faghihi, and Nemazee) affiliated to Shiraz University of Medical Sciences, Shiraz, southern Iran. The study was approved by the Ethics Committee of Shiraz University of Medical Sciences. The inclusion criteria were: 30-70 years of age, no psychological disorder based on a psychologist’s advice, no history of previous PTCA, and lack of any types of speech and hearing disorders. Using the results of a previous study^15^ and the following formula a sample size of 62 patients, 31 in each group, was calculated to detect the differences between the two groups (α=0.05 and 80% power):

$$n=\frac{2\sigma^{2}{\left({\text{Z}}_{\beta }+{\text{Z}}_{\alpha /2}\right)}^{2}}{{\text{d}}^{2}}$$

Considering the probable patient drop out, the number of patients in each group was increased to 40. Initially 228 patients scheduled for PTCA in the mentioned hospitals were screened for participation, among which 123 were ineligible and 25 patients were not interested to take part in the study, coming up with a sample of 80 patients. 

Demographic information of patients, including age, sex, marital status, education level, and occupation, previous angioplasty, was collected through a questionnaire. DASS-21 questionnaire (depression, anxiety and stress scale) was used to measure the patients stress, anxiety, and depression level. This questionnaire’s validity for stress, anxiety, and depression was 76%, 66%, and 70%, respectively^32^ and its reliability was 91%^33^ for the Iranian population.

**Figure 1:**
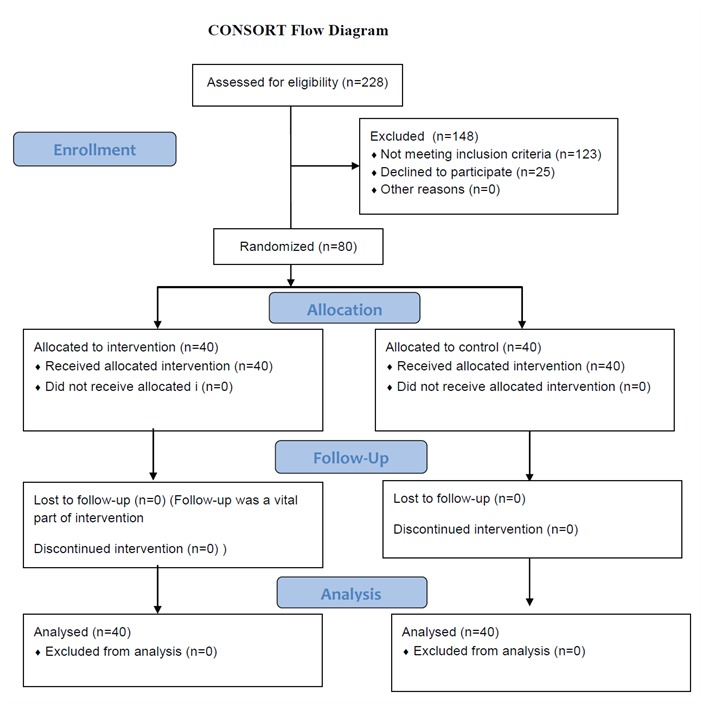
Flow diagram of patients’ progress through the stages of randomized controlled trial

The aim of the study was explained to the participants, and before random assignment, written informed consent was obtained from all the patients. However the participants were allowed to leave the study at any desirable time. Recruited patients were randomly assigned to equal control and experimental groups using the simple randomization method. We randomized weeks in which the discharge plan and the routine plan were to be performed. We numbered the weeks (1.experimental 2.control); then, the sequence of weeks was drawn up by tossing a coin. Figure 1 shows the recruitment of patients and their assignment to the two study groups.

The control group received routine oral instructions on being NPO, shaving, and how to take the medications. And after angioplasty, at discharge, they also received a pamphlet containing information on diet, amount of activities, and incidence of pain and bleeding. The experimental group participated in a 30-minute training session about the reason for angioplasty, its procedures and the required equipment for angioplasty, and the events occurring during angioplasty. At discharge, they received a booklet about the necessity of changes in the lifestyle such as doing exercises, giving up smoking, controlling their stress and anxiety and relaxation, amount of activities, diet, administered drugs and their side effects, when and how to have sexual activity, taking care of the incision and its pain, necessary actions in the case of bleeding and/or hematoma, referral of symptoms, and also necessity of participation in cardiac rehabilitation program. These subjects were explained to the patients and a family member, face-to-face, in a forty-minute session. All the subjects were selected from medicine and nursing textbooks and were confirmed by professors at Fatemeh School of Nursing in Shiraz. The patients were followed up on the phone once a week during two weeks immediately after discharge. DASS-21 questionnaire was also filled in by the patients of both groups to measure their stress, anxiety and depression level. Demographic information of patients, including age, sex, marital status, education level, and occupation, previous angioplasty, was collected through a questionnaire. DASS-21 questionnaire (depression, anxiety and stress scale) was used to measure the patients stress, anxiety, and depression level. This is a standardized questionnaire, according to the studies in Iran the validity for stress 76%, anxiety 66% depression70%^32^ and the  reliability r=91%^33^ was reported.

Data were analyzed using SPSS software, version 18. Multi-sample RMAOV was used to assess the change in stress, anxiety, and depression level over time. Due to a significant interaction between time and groups, we used Student’s t and one-sample AOV test for subgroup analysis. To analyze the demographic data, the Chi-square test was used.

## Results

A total of 80 patients participated in this study (40 in the control and 40 in the experimental groups). The patients’ demographic data are shown in table 1. The control and experimental groups did not differ significantly in terms of demographic characteristics (table 1). Moreover, according to the results of independent t-test the control and experimental groups did not differ significantly in terms of stress (P=0.696), anxiety (P=0.110) and depression (P=0.073). Moreover, comparing the discharge with the admission time, the experimental group showed no significant difference in comparison with the control group. However, the mean differences of the experimental group a month after discharge, as compared with the time of admission, were higher than those of the patients in the control group (P<0.05, table 2). Stress, anxiety and depression in the control group did not reveal a significant difference at discharge compared with the time of admission (P=1.000), while stress (P<0.001), anxiety (P=0.021) and depression (P=0.048) were statistically significant a month after discharge. In the experimental group, we found that stress (P=0.705), anxiety (P=0.205) and depression (P=0.157) reduced upon admission; however, the difference was not significant, whereas a mount later it was statistically significant (P<0.05, table 2).

**Table 1:** Patients’ demographic data

**Variable**		**Control group n=40**			**Experimental group n=40**			**Statistical evaluation**	
		**mean±SD**	**n**	**%**	**mean±SD**	**n**	**%**	**t**	**Chi-Square**
								P value*	**P value**
Age		55±7.9			54±7.6			0.975	
Sex	Male		23	57.5		22	55		0.500
	Female		17	42.5		18	45		
Education level	Illitrate		7	17.5		9	22.5		0.780
	Primary school		22	55		17	42.5		
	Middle school		7	17.5		7	17.5		
	High school		2	5		4	10		
	Higher education		2	5		3	7.5		
Marital status	Married		38	95		39	97.5		0.500
	Single		2	5		1	2.5		

P value*=Independent t tests for age

**Table 2:** Comparison of stress, anxiety and depression in control and experimental groups

Time Variable **mean±SD**		** T_1_**	T_2_	T_3_	P value*	
		**mean±SD**	**mean±SD**	** T_1-_T_2_**	** T_1-_T_3_**	
Stress	Experimental	6.82±4.45	6.40±4.22	3.57±3.50	0.705	<0.001
	Control	8.12±4.42	8.20±4.58	9.55±5.41	1.000	<0.001
	P value	0.194	0.072	<0.001		
Anxiety	Experimental	4.02±2.95	3.40±2.83	1.60±2.42	0.205	<0.001
	Control	4.12±3.09	4.20±3.15	4.82±3.51	1.000	./021
	P value	0.883	0.273	<0.001		
Depression	Experimental	5.00±3.44	4.45±3.31	2.45±2.49	0.157	<0.001
	Control	4.85±3.02	4.97±3.04	5.95±3.76	1.000	0.048
	P value	0.837	0.463	<0.001		

T_1_=Time of admission; T_2_=Time of discharge; T_3_=A month after discharge; P value*=Repeated measurement Anova in the groups. P value= Independent t-tests for between groups.

## Discussion

The objective of this study was to investigate the impact of discharge plan on stress, anxiety, and depression in PTCA. The findings revealed that the mean stress, anxiety, and depression scores of both the experimental and control groups on the day of discharge were not statistically significant; however, the mean stress, anxiety, and depression scores of the experimental group decreased more than that of the control group. In one study, researchers assessed the impacts of preparatory education on recovery following coronary artery bypass grafting (CABG) and found that there was no significant difference between both intervention and control groups at their discharge.^34^ Nonetheless, other researchers investigated the effects of discharge teaching and counseling on the anxiety and depression level of patients undergoing CABG. Their findings suggest that the stress and anxiety of the intervention group on discharge decreased significantly compared with those of the control group; in other words, the difference between these two groups on discharge in terms of stress and anxiety was statistically significant.^13^

Numerous studies suggest that the ailment, hospitalization, complicated medical care, and diagnosis methods will all expose the patients to stressors.^35^ Accordingly, not only the diagnosis of CAD is a life-threatening factor, but also it makes the patients change their lifestyle. Moreover, lack of certainty over the procedure, concerns about return of the symptoms, and emergence of coronary artery events are considered as stressors.^5^ In addition, other factors, such as short length of hospitalization which allows less psychological intervention and training and patient’s concerns on their discharge about their lower ability in performing their duties make their adaptation to the illness more cumbersome.^6^^-^^21^ According to these facts, stress, anxiety and depression of patients in the intervention group, who were provided with training before the procedure and discharge, was lower than that of the control group, but the difference was not statistically significant This is owing to the fact that for their adjustment to the illness, patients require a follow-up and more time. This fact was confirmed by the finding that a month after the procedure and with the two-week follow-up on the phone done by the researcher, stress, anxiety and depression decreased significantly in the experimental group compared with the control group. Different research studies since 1995 have found that after the angioplasty procedure, the patients were extremely concerned about symptoms, treatments and complications, activities, enhancing their quality of life upon discharge and these concerns decreased in the experimental group.^27^^,^^28^

Other researchers evaluated patients with myocardial infarction and suggested that the discharge plan a month after the intervention reduced the patients’ anxiety.^1^ Another study showed that teaching and counseling at discharge lowered the stress and anxiety in patients a month after the intervention.^13^ Moreover, in a study conducted on hospitalized elders with hip fracture due to falling, researchers found that discharge planning one and three weeks after their discharge improved the patients’ mental health.^35^ Another researcher surveyed the impact of a continuous care on stress, anxiety, and depression of hemodialysis patients and found that the discharge planning was effective in improving their life status.^33^ Other researchers investigated the effects of an intervention on illness perception for patients with myocardial infarction and found that intervention on illness perception may alter and improve the rates of return to work in these patients.^36^ Their findings are in the same line with ours and confirm the effectiveness of discharge planning in lowering anxiety and depression. The stress, anxiety and depression a month after discharge increased in the control group, which is in accordance with the findings of another study which suggested that stress and anxiety increased after PTCA^5^^,^^6^ because of the short length of hospitalization in PTCA, due to which the chance for teaching and psychological intervention for patients and family is less probable and it is a challenging issue for them.^16^ Moreover, clinical personnel believe that this procedure is less invasive compared with by–pass surgery; therefore, those patients require less psychological support after the procedure.^14^ Consequently, the researcher came to the conclusion that patients in the control group for whom no discharge plan was conducted received less training and psychological support, in turn, causing an augment in their stress, anxiety and depression. Another study, also, indicated that lack of information and negative illness perceptions are associated with new onset of depression following myocardial infarction^37^ which confirms the augment of stress and depression in the control group.

This study had several limitations. First, the patients participating in the study were mostly illiterate individuals. Further research should be conducted with literate participants and the results can be compared with the current findings. Second, the time period for carrying out this study was short. Besides, chest pain two months after the procedure in some patients might have affected their stress and anxiety. Further research is recommended to investigate stress, anxiety and depression in patients 3 or 4 months after the procedure.

## Conclusion

The findings of this study verify the effectiveness of discharge planning in the reduction of stress, anxiety, and depression in patients undergoing angioplasty. Hence, nurses who are the key members of the clinical team with their critical roles in training and taking care of PTCA can prevent the incidence of psychological problems by planning an appropriate discharge plan in line with the patients’ requirements. This care should begin upon admission and continue to the discharge or even after their discharge. 

## References

[1] Babaei M, Mohammad Khan, Alhani F (2011). Influence of discharge planning on anxiety levels in patients with myocardial infarction. Koomesh.

[2] Negarandeh R, Nayeri ND, Shirani F, Janani L (2012). The impact of discharge plan upon re-admission, satisfaction with nursing care and the ability to self-care for coronary artery bypass graft surgery patients. Eur J Cardiovasc Nurs.

[3] Hadaegh F, Harati H, Ghanbarian A, Azizi F (2009). Prevalence of coronary heart disease among Tehran adults: Tehran Lipid and Glucose Study. East Mediterr Health J.

[4] Alinejad Z (1999). Medical-Surgical of The Heart-A Psychophysiologic Approach.

[5] Gallagher R, Trotter R, Donoghue J (2010). Preprocedural concerns and anxiety assessment in patients undergoing coronary angiography and percutaneous coronary interventions. Eur J Cardiovasc Nurs.

[6] Astin F, Jones K, Thompson DR (2005). Prevalence and patterns of anxiety and depression in patients undergoing elective percutaneous transluminal coronary angioplasty. Heart Lung.

[7] Dehdari T, Heidarnia A, Ramezankhani A (2008). Anxiety, self efficacy expectation and social support in patients after coronary angioplasty and coronary bypass. Iranian Journal of Public Health.

[8] Gallagher R, Trotter R, Donoghue J (2010). Preprocedural concerns and anxiety assessment in patients undergoing coronary angiography and percutaneous coronary interventions. Eur J Cardiovasc Nurs.

[9] Lett HS, Blumental JA, Babyak MA (2004). Depression as a Risk Factor for Coronary Artery Disease: Evidence, Mechanisms, and Treatmetn. Psychosomatic Medicine.

[10] Wells KB, Golding JM, Burnam MA (1989). Affective, substance use, and anxiety disorders in persons with arthritis, diabetes, heart disease, high blood pressure, or chronic lung conditions. General Hospital Psychiatry.

[11] Carney RM, Freedland KE, Miller GE, Jaffe AS (2002). Depression as a risk factor for cardiac mortality and morbidity: A review of potential mechanisms. Journal of Psychosomatic Research.

[12] Roohafza H, Sadeghi M, Attari A, Afshar H (2007). Serum lipids in patients with mixed anxiety derpressive disorders. The Journal of Qazvin Univ of Med Sci.

[13] Cebeci F, Çelik SŞ (2011). Effects of discharge teaching and counselling on anxiety and depression level of CABG patients. Turkish J Thorac Cardiovasc Surg.

[14] Barefoot JC, Brummett BH, Helms MJ (2000). Depressive Symptoms and Survival of Patients With Coronary Artery Disease. Psychosomatic Medicine.

[15] Moser DK (2007). “ The Rust of Life”: Impact of Anxiety on Cardiac Patients. American Journal of Critical Care.

[16] Mayou RA, Gill D, Thompson DR (2000). Depression and anxiety as predictors of outcome after myocardial infarction. Psychosomatic Medicine.

[17] Lane D, Carroll D, Ring C (2000). Effects of depression and anxiety on mortality and quality-of-life 4 months after myocardial infarction. Journal of Psychosomatic Research.

[18] Lane D, Carroll D, Ring C (2001). Mortality and quality of life 12 months after myocardial infarction: effects of depression and anxiety. Psychosomatic Medicine.

[19] Lane D, Carroll D, Ring C (2001). Predictors of attendance at cardiac rehabilitation after myocardial infarction. Journal of Psychosomatic Research.

[20] Sullivan MD, LaCroix AZ, Spertus JA, Hecht J (2000). Five-year prospective study of the effects of anxiety and depression in patients with coronary artery disease. American Journal of Cardiology.

[21] Duogas B (1992). Principle patient care, comprehensive theory to nursing.

[22] Vanaki Z, Habibipoor B (2009). The effect of discharge planning on patient satisfaction. J Hamedan Uni Med Sci.

[23] Makaryus AN, Friedman EA (2005). Patients understanding of their treatment plans and diagnosis at discharge. Mayo Clin Proc.

[24] Forster AJ, Murff HJ, Peterson JF (2003). The incidence and severity of adverse events affecting patients after discharge from the hospital. Annals of Internal Medicine.

[25] Forster A, Clark H, Menard A (2004). Adverse events among medical patients after discharge from hospital. CMAJ.

[26] Taylor C, Lillis C (1996). Fundamental of nursing.

[27] Corones K, Coyer FM, Theobald KA (2009). Exploring the information needs of patients who have undergone PCI. British Journal of Cardiac Nursing.

[28] Kattainen E, Merilainen P, Jokela V (2004). CABG and PTCA patients’ expectations of informational support in health- related quality of life themes and adequacy of information in 1-year follow-up. Eur J Cardiovasc Nurs.

[29] Yohannes AM, Yalfani A, Doherty P, Bundy C (2007). Predictors of drop-out from an outpatient cardiac rehabilitation programme. Clin Rehabil.

[30] Januzzi Jr, Stern TA, Pasternak RC, DeSanctis RW (2000). The influence of anxiety and depression on outcomes of patients with coronary artery disease. Archives of Internal Medicine.

[31] Yalfani A, Nazemi F, Safi R, Jorge M (2012). The effect of exercise rehabilitation on Anxiety and depression in patients afte coronary artery bypass graft. Sci J Hamadan Univ Med Sci.

[32] Sahebi A, Asghari MJ, Salari R (2005). Validation of depression, anxiety and stress (DASS-21) for Iranian population. Iranian Psychol.

[33] Aghebati N (2005). Effects of touch theraphy on pain, depression, anxietyand stress of hospitalized patients with cancer dissertation.

[34] Shuldham CM, Fleming S, Goodman H (2002). The impact of pre-operative education on recoveryfollowing coronary artery bypass surgery. A randomized clinical trial. European Heart Journal.

[35] Huang TT (2005). A randomized clinical trial of the effectiveness of a discharge planning intervention in hospitalized elders with hip fracture due to falling. Journal of Clinical Nursing.

[36] Broadbent E, Ellis CJ, Thomas J (2009). Further development of an illness perception intervention for myocardial infarction patients: a randomized controlled trial. J Psychosom Res.

[37] Dickens C, McGowan L, Percival C (2008). Negative illness perceptions are associated with new-onset depression following myocardial infarction. Gen Hosp Psychiatry.

